# Elementary Students Prefer Extra Reading Practice Before Reading in Front of Peers

**DOI:** 10.1007/s40617-025-01099-0

**Published:** 2025-09-22

**Authors:** Kristen L. Newell, Scott P. Ardoin, Jeanne M. Donaldson

**Affiliations:** 1 Department of Psychology, Louisiana State University, 236 Audubon Hall, Baton Rouge, LA 70803-5501 USA; 2 Department of Educational Psychology, University of Georgia, Athens, GA USA

**Keywords:** Active student responding, Literacy, Oral reading fluency, Preference, Reading intervention, Repeated reading

## Abstract

We used a multi-element design to determine student preferences for the arrangement of a repeated reading intervention, as either preparation for (i.e., pre-teaching) or review of reading material, and how these arrangements affected their likelihood of volunteering to read during small-group sessions. Three fourth-grade students, identified by their teacher as behind in reading proficiency, participated in small-group reading sessions together. We used a concurrent chains arrangement to assess preference for repeated reading timing. During the choice phase, all three participants selected the pre-teaching condition most frequently. During small group sessions, all three participants consistently volunteered to read their practiced pre-teaching passage at almost every opportunity. For all three participants, words read correctly per minute was higher during small group for the pre-teaching passage that was practiced during the morning intervention session in comparison to other conditions and other passages in the pre-teaching condition across all phases. These findings are consistent with literacy research demonstrating that repeated reading improves oral reading fluency for practiced passages and indicate that using repeated reading as a pre-teaching strategy enhances students’ willingness to volunteer to read aloud. Additionally, these findings suggest that students prefer to practice before reading in a group setting.

**Supplementary Information:**

The online version contains supplementary material available at https://doi.org/10.1007/s40617-025-01099-0.

Students with literacy deficits are significantly more likely to exhibit challenging behaviors, reduced task engagement, and poor self-control (Morgan et al., 2008a, 2008b). They also often avoid reading, which can result in them falling further behind their peers in reading skills and academic achievement (Guthrie et al., 2022). Furthermore, students with reading difficulties may contact punishment when reading in front of peers during class. For example, a struggling reader who is called upon to read a passage during class may be ridiculed by peers for reading slowly or corrected by their teacher for mispronouncing a word. In turn, this pairing of punishment and reading aloud can result in a conditioned aversion to participating in class reading, thus reducing the student’s engagement and increasing avoidance and escape behaviors surrounding reading. Studies indicate this scenario frequently occurs among struggling readers (Fishstrom et al., 2024; Grills-Taquechel et al., 2011).

Reading difficulties can result in children experiencing a multitude of socio-emotional problems, including peer rejection/neglect, negative emotional affect, and social skills deficits (Grills-Taquechel et al., 2011). Furthermore, children who struggle to read are more likely to experience symptoms of depression, anxiety, and school avoidance in comparison to students who are strong readers (Grills et al., 2022). In a study involving 536 students with reading difficulties, children who struggled with reading comprehension, word reading fluency, and text reading fluency demonstrated higher levels of anxiety in comparison to children with typical literacy development (Fishstrom et al., 2024). Reducing anxiety surrounding reading can lead to increases in class engagement and confidence and decreases in anxiety levels, depression, and withdrawal for struggling readers (Francis et al., 2021).

Students’ engagement with learning activities during class increases their likelihood of academic success (Lei et al., 2018), including achieving higher grades and being less likely to drop out of school (Klem & Connell, 2004). Academic engagement can refer to a multitude of specific classroom behaviors, such as reading aloud or silently, discussing lesson content, asking and answering questions, and writing (Greenwood et al., 2002). One strategy that may promote class engagement, and thus support reading skill development, is for teachers to provide opportunities for active student responding. Active student responding occurs when a student emits an observable response to classroom instructions, such as raising their hand to answer a question, reading aloud, engaging in choral responding with the class, or writing an answer on a response card (Heward et al., 1989; Narayan et al., 1990). By simply arranging opportunities for active student responding during class, teachers can increase their students’ engagement with academic tasks. Student academic achievement is correlated with opportunities for active student responding, and it is associated with increasing a variety of critical academic skills (Alber-Morgan, 2006; Narayan et al., 1990).

However, providing opportunities for active student responding may be necessary but not sufficient to improve skills for students lacking the prerequisite skills to respond correctly. That is, more opportunities to respond incorrectly is unlikely to further skill development. Furthermore, active student responding may contact punishment (e.g., error correction from the teacher or peer ridicule) if a student responds with an incorrect answer, leading to more anxiety surrounding overt responding in class. For students with reading difficulties, disengagement and off-task behavior are common ways to avoid reading and the stressors associated with it (Sukovieff & Kruk, 2021). Given that students with poor reading ability are at greater risk of generalized anxiety disorder, significant school avoidance, and depression (Lerthattasilp et al., 2023), increasing reading skills and reducing negative associations with reading is a matter not only of literacy but also of mental health.

One intervention for which there exist multiple studies demonstrating beneficial effects on students’ academic engagement and skills is repeated reading. Repeated reading involves a student re-reading a passage either a pre-specified number of times or to a pre-established reading rate (Begeny et al., 2009). When provided with opportunities for repeated reading, students’ reading fluency and comprehension is enhanced (Ardoin et al., 2016; Begeny et al., 2009; Therrien, 2004). Additionally, research has shown that repeated reading can produce an increase in students’ on-task behavior during reading instruction (Scott & Shearer-lingo, 2002). Therrien and Kubina (2006) recommend that the appropriate performance criterion for repeated readings, defined as the number of words read correctly per minute, should be selected on the basis of the student’s current reading level and should allow for the student to achieve the criterion in a reasonable amount of time. For example, appropriate performance criteria may be 94 correct words per minute for a second-grade student and 114 correct words per minute for a third-grade student (Therrien & Kubina, 2006). Repeated reading is frequently included in comprehensive interventions for reading (e.g., Peer-Assisted Learning Strategies, Fuchs et al., 1997). However, to our knowledge, no prior studies have determined the effects of repeated reading interventions received during tutoring sessions on (a) volunteering to read during small group instruction, or (b) student preference for practicing reading materials via repeated reading before (i.e., pre-teaching) or after (i.e., review) being asked to read in front of peers.

Pre-teaching involves the advanced introduction of content in order to prepare students for upcoming classroom instruction (Munk et al., 2009). One example is vocabulary pre-teaching, which involves introducing new vocabulary words and definitions from an upcoming lesson to the students. Pre-teaching can improve test scores and grades for students by introducing necessary component skills prior to introducing an academic task (Munk et al., 2009). Burns et al. (2004) found that pre-teaching unknown keywords increased reading fluency and comprehension in elementary school students with reading disabilities. When used as a pre-teaching strategy, repeated reading may involve a student repeatedly reading material for the upcoming lesson until they reach a certain mastery criterion. This approach increases the likelihood that the student will read the passage with fluency and accuracy, thereby increasing the probability of reinforcement through positive feedback or praise, while reducing the chances of errors and the associated possibility of punishment or aversive consequences (Ardoin et al., 2018). Pre-teaching can be contrasted with the common practice of content review, in which content is repeated or clarified after it has been presented in the lesson. Reviewing information can occur during or outside of class (e.g., homework, special education pull-out services, supplemental tutoring). As a review strategy, repeated reading may involve the student re-reading passages read during a previous classroom lesson until the student reaches a specified criterion.

Both pre-teaching and review strategies may have specific strengths and weaknesses for promoting reading fluency and confidence to read in front of peers, and assessing student preference is an important aspect of determining the social validity of the procedures. One way to assess preference is to offer students choices of what they will experience using a concurrent chains schedule (Hanley, 2010). Using a concurrent chains procedure to assess preference allows the participant to engage in self-determination and play an active role in the planning process (Hanley, 2010). Furthermore, including the participants’ preferences for intervention can increase responding, improve task performance, and reduce levels of disruptive behavior (e.g., Dunlap et al., 1994). Additionally, when given the option to take ownership of the interventions they experience, students consistently select to do so (Peltier et al., 2023).

The primary purpose of this study was to examine how the arrangement of repeated reading, either as preparation for or review of reading material, affects volunteering to read when students participated in small-group reading sessions with peers. Additionally, we examined student preference for whether repeated reading was done in preparation for reading in front of peers or as a review of reading material previously read in front of peers.

## Method

### Participants, Setting, and Materials

The research team recruited students from an elementary school in the southern United States. The fourth grade English Language Arts teacher expressed an interest in having students participate in a study to improve their reading fluency. She was asked to nominate three students with reading difficulties and low rates of active responding during reading instruction who regularly attended school. All nominated participants’ caregivers provided permission for their child to participate. An experimenter acquired participant assent at the commencement of the study by describing the study to the students, allowing students to ask questions, and asking if they would like to participate. Participant assent was also acquired each session by asking the student if they wanted to read with the experimenter/small group that day. Participants assented at every opportunity. Participants consisted of three, fourth-grade African American girls who did not have any disability diagnoses. Rachel and Chelsea were 9 years old, and Riley was 10 years old. The study took place at a local elementary school where 87% of the enrolled students were African American, and 97% were eligible for free lunch.

An experimenter implemented an individual intervention session with each participant, as well as leading small-group sessions comprising the three participants. Individual intervention sessions and small group sessions each were conducted once per day, 3 to 5 days per week. Individual sessions occurred in the morning in a private office in the school library. Small group reading sessions, during which academic engagement was measured, were conducted in the afternoon, approximately 4 hours after the individual intervention sessions, in the same private office.

To reduce the time burden on the teacher, repeated reading passages were selected by the researcher from a list of books on second and fourth grade reading levels, based on the Scholastic Guided Reading Program Levels (Fountas & Pinnell, n.d.-b). Passage difficulty was further confirmed using an online Flesch-Kincaid Grade Level calculator (Scott, n.d.). We initially selected fourth-grade reading passages because all participants were in fourth grade and would be expected to read materials at this level in class. After the first choice phase, we used second-grade reading passages to better align with participants’ reading level. For individual pre-teaching sessions, previewing procedures were provided on the passages to be read during that afternoon’s small group session. For individual review sessions, passages included reading material used in the most recent small group session, which was typically the prior day but varied between 1 and 21 days (*M* = 5 days*, mode* = 1 day) due to absences, weekends, and holidays. Additional materials included a timer and a variety of small prizes (e.g., stickers, erasers, squishy ball) that children were allowed to select and keep after each session.

### Response Measurement and Interobserver Agreement

Trained graduate and undergraduate research assistants collected data using a data sheet that was embedded into the printed reading passage. The primary dependent variable was the frequency of condition selection responses, which provided an indication of each participant’s intervention preference. Opportunities to select the condition they would experience occurred at the start of individual intervention sessions during the choice condition, and verbal reports of the most preferred condition occurred at three time points dispersed across the study (i.e., prior to the participants experiencing either repeated reading intervention and only receiving a description of the conditions, after the participants had experienced two sessions of each repeated reading intervention, and prior to beginning the choice condition). During the verbal preference assessment, the experimenter described each condition to the participant, asked which condition they thought they would like best, and recorded the participant’s response. The verbal preference assessments were conducted immediately before the start of the morning intervention sessions on the days they were administered. During the choice conditions, a selection response occurred when the participant indicated which procedure they would experience by either touching the card with the corresponding color and name (see procedure below) or saying the condition name. A verbal report of the most preferred condition occurred when the student stated which of the three conditions (e.g., baseline, pre-teaching, or review) they preferred when asked by the experimenter.

Research assistants collected data on several secondary dependent variables. During individual intervention and small group sessions, data were collected on words read correctly per minute (WRCM). During small group sessions, data on the percentage of volunteering to read were collected. During the individual intervention, WRCM was measured by recording the total number of words read for each attempt and subtracting errors. During small group, WRCM was measured by recording the number of WRCM from each passage read and then averaging those scores. The percentage of volunteering to read was determined by dividing the frequency of volunteering to read by the frequency of opportunities to read. An opportunity to read was defined as the experimenter asking if there was any student willing to read and volunteering to read was defined as any time a student raised their hand indicating their willingness to read. Each participant was given 10 opportunities to volunteer to read during the afternoon small group.

Research assistants also recorded the frequency of errors and percentage of words accurately read during each participant’s small group readings, and the percentage of volunteering to answer comprehension questions. Accuracy was measured by dividing the total number of words read correctly by the total number of words read. Mean error frequency was measured by calculating the number of errors made during each reading passage in the small group and averaging the results. During the individual intervention session, error frequency was measured by recording the total number of errors made in the 1-min reading passage. Volunteering to answer comprehension questions was defined as any time a participant raised their hand to answer a question posed by the experimenter, regardless of whether the participant was called on, and was calculated using the same measurement as volunteering to read. Each participant was given six opportunities to volunteer to answer comprehension questions during the afternoon small group.

A secondary observer collected data independently on all dependent variables to calculate interobserver agreement. Agreement on volunteering to read, WRCM, product measures, and single-opportunity responses (selection responses in the choice condition and verbal preference assessment) were calculated using point-by-point exact agreement. Secondary data were collected across all conditions across all participants for a mean 71.7% (range, 43–100%) of sessions. Volunteering to read IOA was 100% for all participants. Mean agreement on WRCM across all conditions for all participants was 98.6% (range, 97.2–99.5%). IOA for selection responses in verbal preference assessment and choice condition was 100% for all participants.

### Experimental Design and Procedure

A multielement design was used to determine the effects of the timing (i.e., pre-teaching or review) of repeated reading on participants’ behavior during small group reading. Intervention and baseline sessions occurred at a 4:1 ratio; that is, pre-teaching and review sessions each occurred twice before a baseline session. After differentiation in WRCM was observed with fourth-grade reading level passages, participants experienced a choice condition. Following the choice condition, procedures were replicated with second-grade reading level passages to better align with participants’ reading level.

**Fig. 1:**
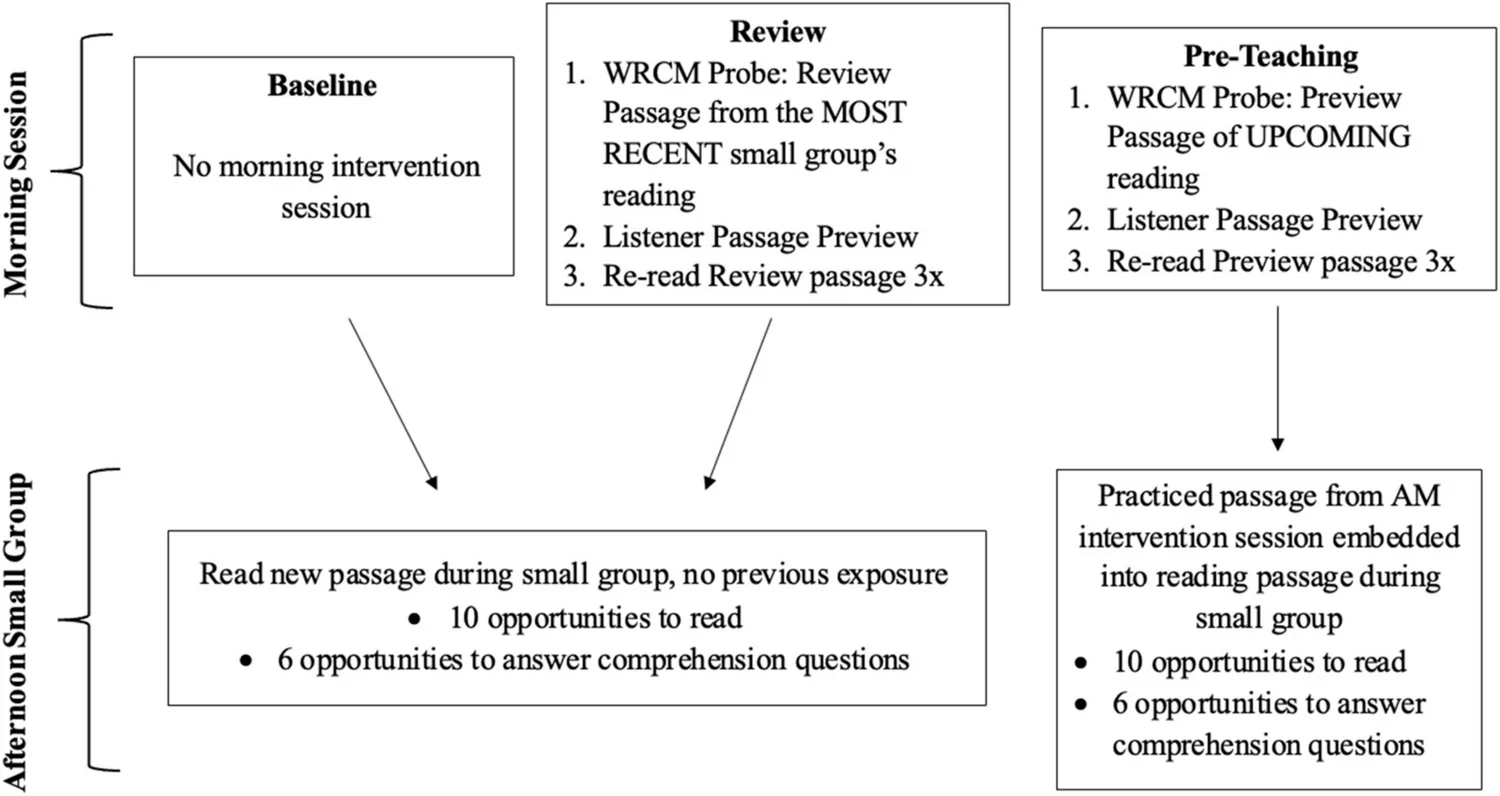
Flowchart of morning and afternoon sessions during each condition. *Note. WRCM* Words read correctly per minute

During each small group session, participants were all provided with a copy of the passage they would be expected to read during that session. The experimenter provided each participant with 10 opportunities to read by asking, “Who wants to read next?” and calling on one of the participants who raised their hand to read. If no participants had raised their hand to read, the experimenter would have read the passage aloud and again asked for volunteers, but this never occurred because at least one child always volunteered for each opportunity. After reading for a minute, the participant was told to stop reading and the researcher provided the next opportunity to read. The experimenter avoided calling on the same participant twice in a row if another participant volunteered to read. When the story was finished, the experimenter asked six comprehension questions related to the reading passage. The group ended after the experimenter provided each participant with 10 opportunities to read and the students answered the six comprehension questions (data for volunteering to answer comprehension questions are available in Supplemental Material). Each participant selected a small incentive or brief activity before returning to class. Figure 1 displays a flowchart of what participants experienced during the morning individual and afternoon group sessions in each condition.

#### Baseline

During baseline session days, participants experienced only small-group reading (i.e., no individual sessions were conducted on that day). The participants received no other reading intervention beyond what was provided by their regular classroom curriculum.

#### Repeated Reading

The experimenter conducted repeated reading sessions with each participant individually in the morning. Both types of repeated reading sessions (i.e., pre-teaching and review) began with a WRCM probe, followed by the experimenter reading the selected passage aloud to the student while the participant silently followed along with their own copy (i.e., listening passage preview; Begeny et al., 2009). The participant then re-read the same passage aloud to the experimenter three times or until they met a mastery criterion based on benchmark WRCM scores for fourth grade (Therrien, 2004). However, no participant ever met the mastery criterion.

When participants misread a word, paused for 3 s, or otherwise indicated that they did not know a word the experimenter modeled the correct pronunciation of the word. At the end of the session, the participant was allowed to select a small item or brief activity before returning to class.

##### Pre-Teaching

During the pre-teaching condition, a green paper with the word “Preview” was on the table as the corresponding stimulus indicating the condition. When the experimenter introduced the condition, they stated that the day would be a “Preview” day, indicated by the green card, while pointing to the card. Prior to the initial WRCM probe, the experimenter stated the following pre-teaching instructions: “This is from the story that you will read again in small group today. First, you are going to read this story to me for 1 minute. Then, it will be my turn to read it to you. Make sure to follow along using your finger, because after I read it, you are going to have to read it back to me three times or until I tell you to stop. Try your best!” During the afternoon small group session on pre-teaching days, if the participant raised their hand to read the passage that they had practiced during the morning’s pre-teaching intervention (i.e., practiced pre-teaching passage), the experimenter selected that participant to read aloud their practiced passage.

##### **Review**

During the review condition, a purple paper with the word “Review” was on the table. When the experimenter introduced the condition, they stated that today would be a “Review” day, indicated by the purple card, while pointing to the card. Prior to the initial WRCM probe, the experimenter stated the following pre-teaching instructions: “This is from a story that you have already read in small group. First, you are going to read this story to me for 1 minute. Then, it will be my turn to read it to you. Make sure to follow along using your finger, because after I read it, you are going to have to read it back to me three times or until I tell you to stop. Try your best!”

#### Choice

Choice sessions were arranged as a concurrent chains preference assessment whereby participants selected a study condition and then experiencing their selection. Before individual intervention sessions, the experimenter placed each colored paper with the condition name in front of the participant, including a baseline option, and described what each paper represented by stating, “If you select the red paper, that means we will not have reading practice today. If you select the green piece of paper, it means that you will read a new passage that you will read again today during group. If you select the purple piece of paper, it means that you will read a passage that you have already read before in group.” The selected condition was then implemented during the individual intervention session. The choice phase concluded when the participant selected the same condition four consecutive opportunities or following 10 total choice sessions.

### Procedural Fidelity

An observer collected data on the extent to which the procedures were implemented as stated on the procedural fidelity checklist during 100% of sessions. A second observer collected the same data during 61% of sessions. We calculated procedural fidelity using point-to-point exact agreement by dividing the number of steps performed correctly by the total number of steps. Checklists consisting of 7 steps for small group sessions and 13 steps for intervention sessions were used to determine the extent to which the experimenter arranged the prescribed session materials and delivered the correct antecedents and consequences. The checklists are available in Supplemental Materials. Procedural fidelity was 100% across all conditions for all participants. IOA for procedural fidelity was 100% across all conditions and participants.

## Results

**Fig. 2:**
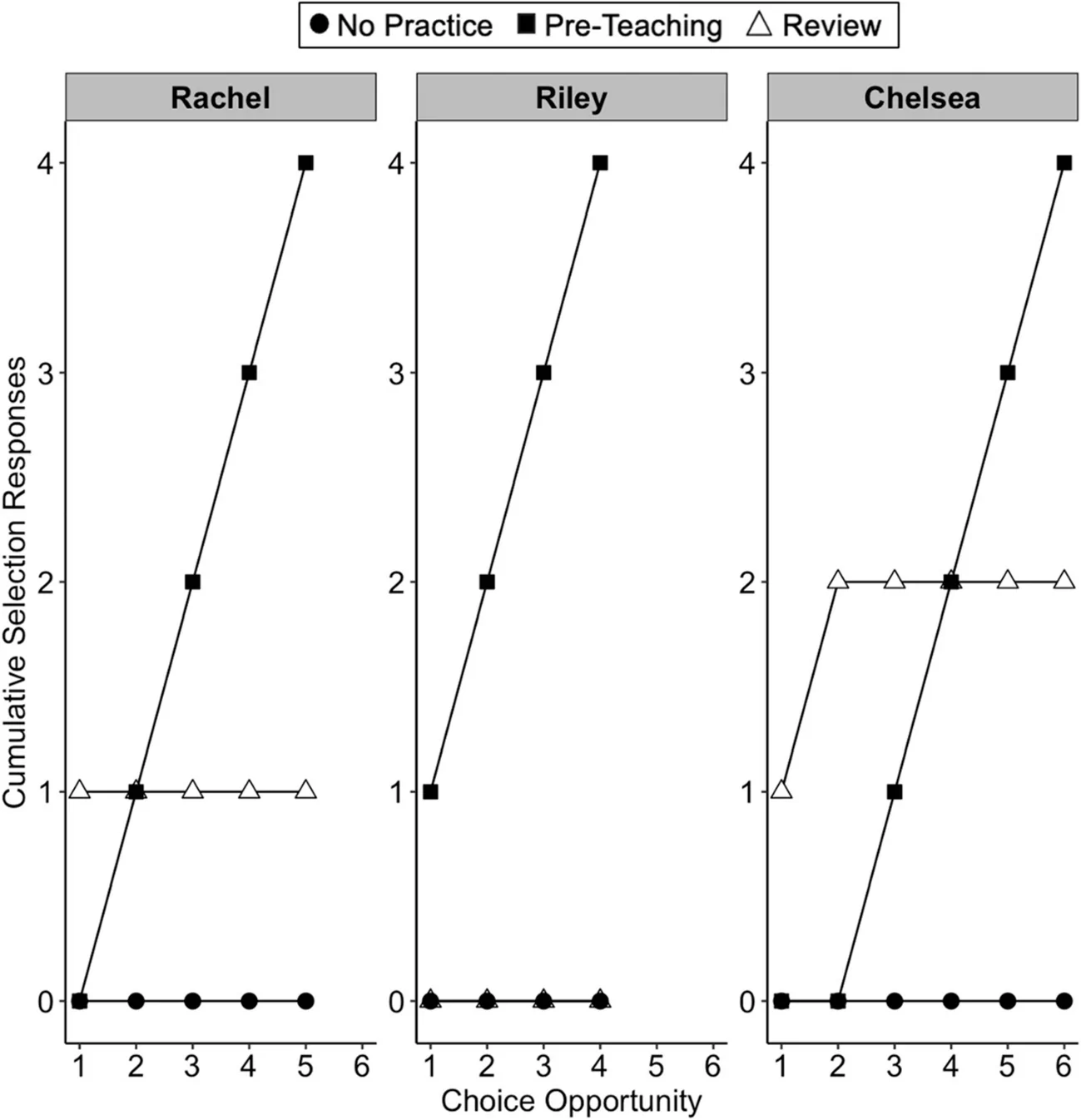
Cumulative selection responses for each condition during the choice phase for all three participants

Figure 2 shows the cumulative selection responses of each condition during the choice phase for all three participants and illustrates that all participants selected the pre-teaching condition most frequently. Further, all participants selected the pre-teaching condition at each of the three time points the verbal preference assessment was administered. No participant selected baseline (“no practice”) on any choice opportunity.

**Fig. 3:**
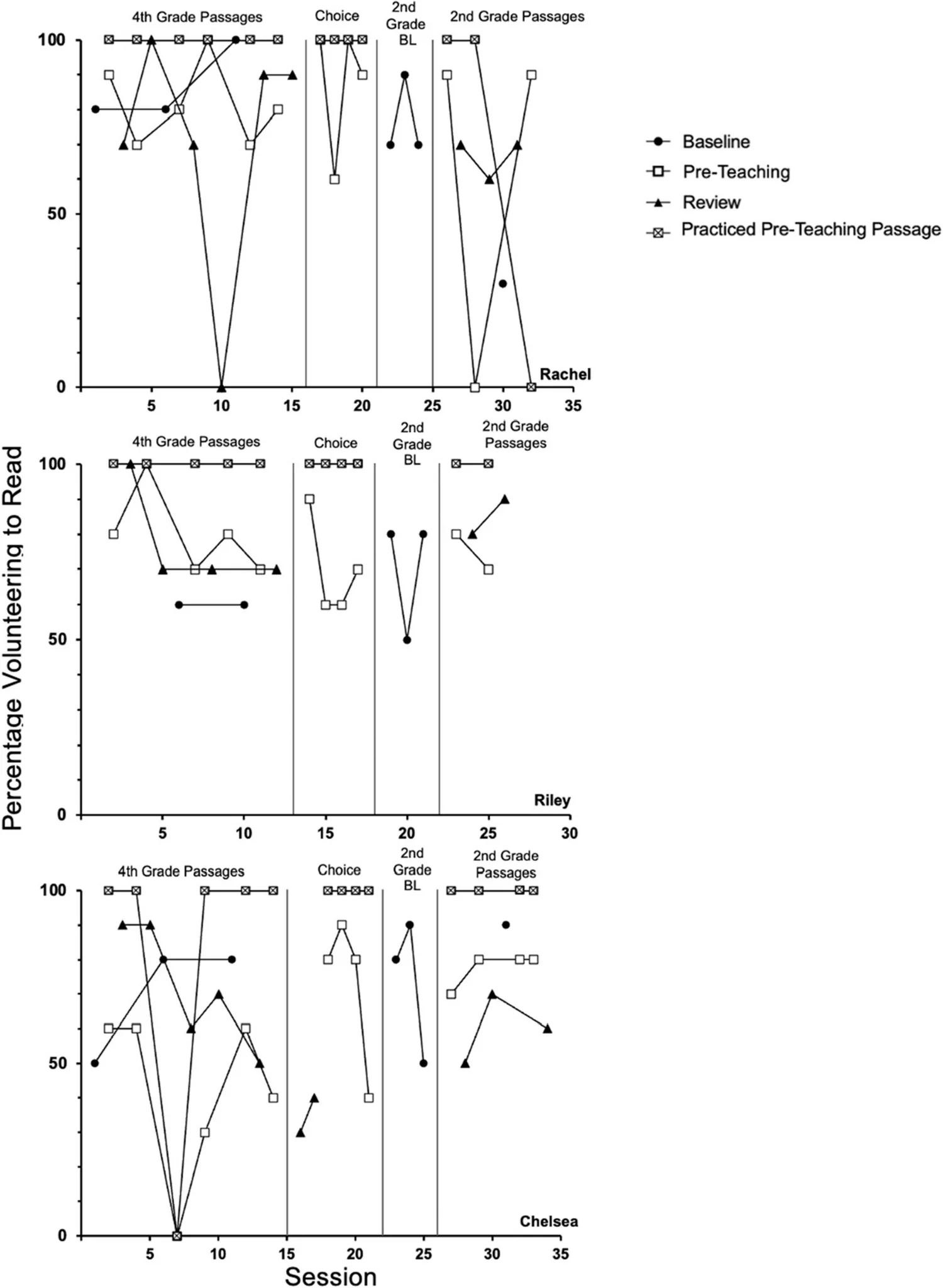
Percentage of volunteering to read during small group sessions for all three participants in all conditions*. Note. BL* baseline

Figure 3 shows the percentage of volunteering to read during small group sessions for all three participants in all conditions. Overall, high rates of volunteering to read occurred for all participants across all conditions. However, the participants consistently volunteered to read their practiced pre-teaching passage at almost every opportunity. Rachel volunteered to read her practiced pre-teaching passage each opportunity, with just one missed opportunity across all sessions. In the other conditions, Rachel’s volunteering to read was more variable, with percentages ranging from 0% to 100% (preview range, 0–100%, *M* = 78.46%; review range, 0–100%, *M* = 68.88%; baseline range, 30–100%, *M* = 74.39%). Riley volunteered to read her practiced pre-teaching passage 100% of opportunities. Additionally, she had high rates of volunteering to read across all conditions (preview range, 60–100%, *M* = 75.45%; review range, 70–100%, *M* = 80%; baseline range, 50–80%, *M* = 64%). Chelsea volunteered to read her practiced pre-teaching passage each opportunity but one. For the other conditions, Chelsea’s volunteering to read was variable with no systematic differences (preview range, 0–90%, *M* = 59.23%; review range, 30–90%, *M* = 61%; baseline range, 50–90%, *M* = 74.29%).

**Fig. 4:**
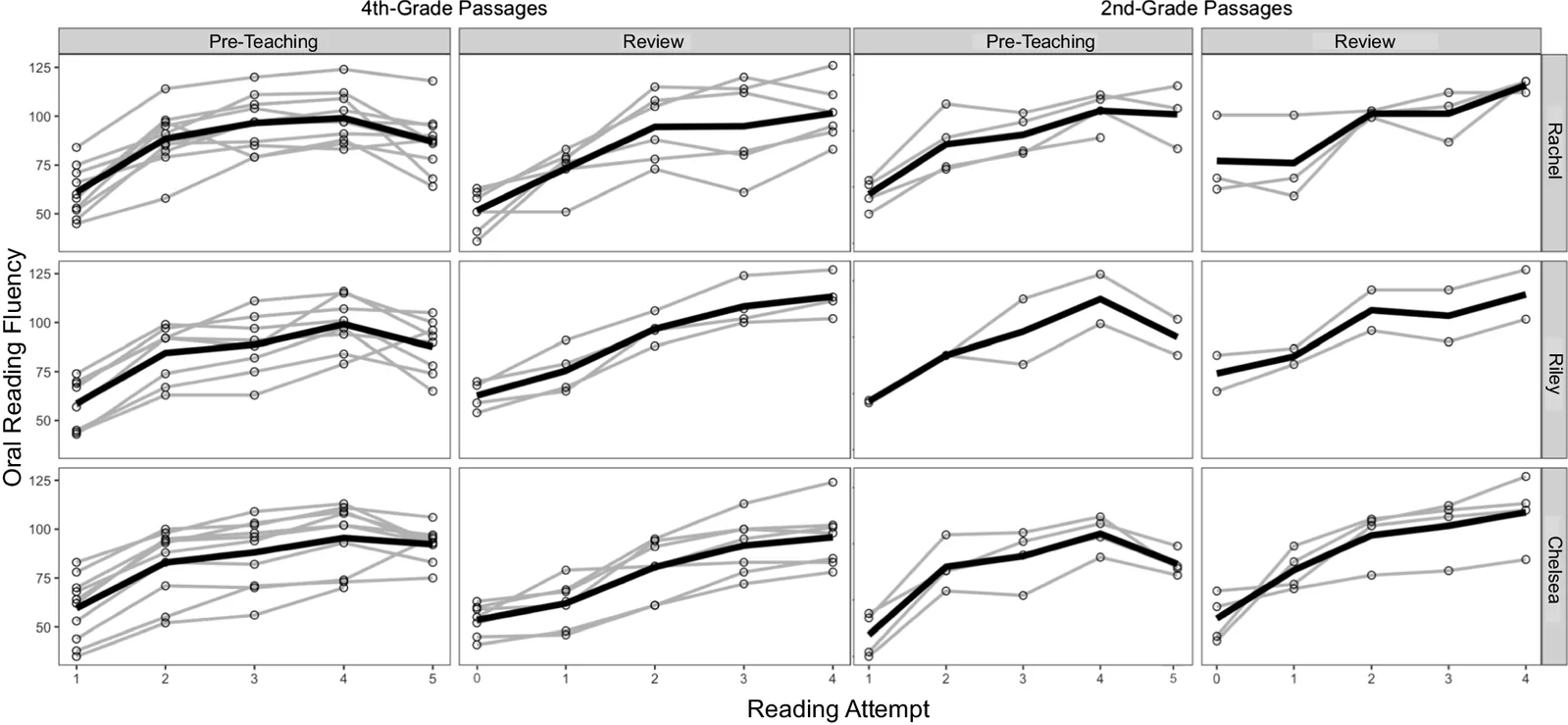
Words read correctly per minute (WRCM) during the morning probes during the pre-teaching and review conditions for each participant. Thick black lines indicate the mean WRCM. Data points at 0 on the x-axis in the review condition indicate reading the passage during afternoon small group prior to reading it during the individual intervention session. Numbers 1 through 4 on the x-axis represent repeated reading attempts during the individual intervention sessions. Data points at 5 on the x-axis in the pre-teaching condition represent a reading attempt of that same passage during small group (practiced pre-teaching passage)

Figure 4 shows the repeated reading WRCM of each participant during the morning probes during the pre-teaching (left panels) and review (right panels) conditions, separated by second and fourth grade reading passages. For all participants, the mean WRCM, represented by the bold black line, was lowest on the first reading attempt and highest on the fourth reading attempt for both grade-level readings. However, the largest gain in WRCM between readings was from attempt 1 to attempt 2 for all participants and in both conditions and grade levels. Of note, listener passage preview occurred between attempts 1 and 2, during which the experimenter read the passage aloud to the participant, providing a model of the correct pronunciation for each word. Following attempt 2, increases in the mean WRCM were not as large as the increases between the initial two attempts. All participants consistently had higher WRCM on attempt 1 in comparison to attempt 0 in the review condition.

**Fig. 5:**
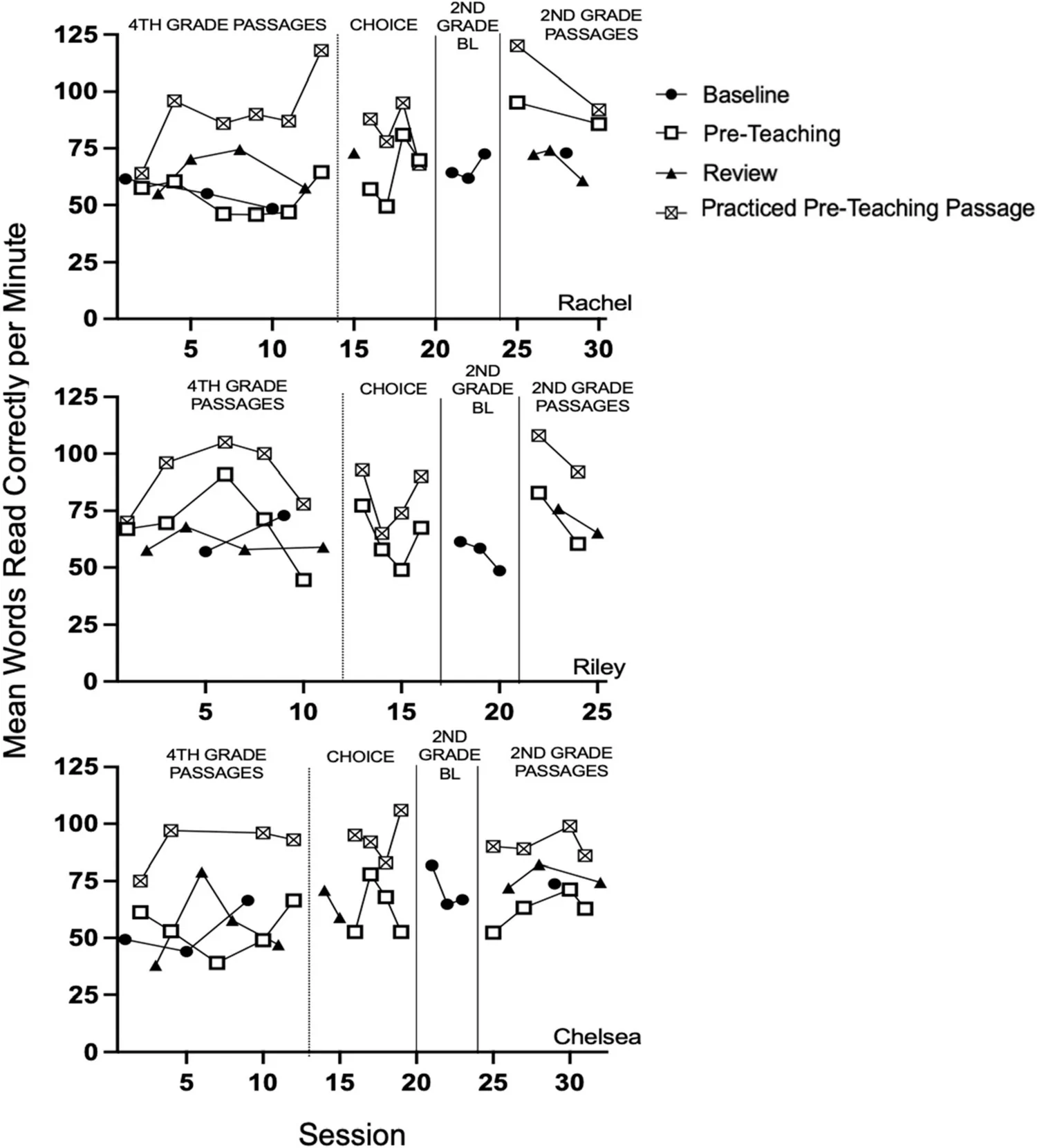
Mean words read correctly per minute (WRCM) during small group for all three participants across sessions. *Note. BL* baseline

Figure 5 shows the WRCM during small group for all three participants across sessions. Pre-teaching passage data points represent the WRCM in small group for only the specific passage that participants practiced in their individual intervention session during the pre-teaching condition. For all three participants, WRCM was higher during small group for the pre-teaching passage that was practiced during the morning intervention session in comparison to other conditions and other passages in the pre-teaching condition across all phases. During fourth-grade and choice phases, there was no consistent difference between pre-teaching, review, and baseline conditions for all three participants. For Rachel and Riley, no systematic differences in average WRCM between pre-teaching, review, and baseline emerged given the limited number of data points. For Chelsea, mean WRCM was higher during the second-grade reading level review condition in comparison to pre-teaching and baseline.

Accuracy and error frequency during the first WRCM probe administered during individual intervention sessions and small group readings are available in Supplemental Materials, as is the percentage of volunteering to answer comprehension questions during the small group.

## Discussion

We evaluated student preference for the timing of a repeated reading intervention, as either pre-teaching upcoming material or review of previously read material, as well as effects on volunteering to read in front of a small group of peers and WRCM. All three participants selected the pre-teaching condition most frequently during the choice condition and exclusively during each of the three verbal preference assessments. Additionally, all participants volunteered to read the passage they practiced that morning during the pre-teaching condition more consistently than passages in any other condition and other pre-teaching passages. Participants’ WRCM while reading the pre-teaching passage practiced the same morning was also consistently higher than their WRCM for any other passage. Lastly, during the individual repeated reading intervention sessions, the largest gain in WRCM between readings was from the first to second attempt for all participants. The results of this study provide valuable insights into the effects of the arrangement of repeated reading and student preference for the intervention within the context of small-group reading sessions.

When participants were asked why they preferred the pre-teaching condition, Rachel stated, “Practicing in the morning makes me feel strong and helps me get ready to read the hard words in group.” Riley reported, “I like practicing in the morning because I know what words I missed, and I won’t get them wrong when I read in front of everyone in the afternoon.” Lastly, Chelsea reported, “I feel more confident when I practice reading with you before group.” Taken together with the verbal preference assessment and selection responses, these results indicate that the participants had a clear preference for the pre-teaching condition, which allowed them to practice what would be read in front of their peers during small group, over reviewing material or not practicing at all. Peer relationships also appeared to have an influence on volunteering to read during small groups and may provide an explanation for the few particularly low data points. Notably, Rachel did not volunteer to read during small group sessions 10 and 28 and reported that she was in an argument with Chelsea that afternoon. Similarly, Chelsea did not volunteer to read during afternoon session 7 and reported that she and Rachel were having a conflict. Despite the children’s general belief that pre-teaching helped them perform better, the review condition led to better initial performance on the morning probe, and pre-teaching led to better performance on the same passage during small group. Thus, the children’s preferences were not necessarily indicative of better oral reading performance in the presence of someone else; both conditions produced better reading performance at different times during the day. However, students specifically preferred the pre-teaching condition that allowed them to perform better in front of their peers during the afternoon small group. It is important to note that the generalizability of these findings is limited by the context in which the intervention was implemented (i.e., outside of the classroom and by an experimenter, rather than a teacher). Future research on this topic could examine student preference for the arrangement of the repeated reading intervention within the classroom setting. Additionally, future research could assess the effects of the arrangement of the intervention on reading comprehension and willingness to answer comprehension questions in the classroom.

These findings provide evidence to support incorporating pre-teaching strategies into existing classroom procedures. Such an approach could facilitate improvements in student performance, especially for struggling readers who benefit from increased confidence and fluency when practicing passages in advance. Although the intervention was studied with individual students in a small-group format, a class-wide adaptation could be beneficial. For example, teachers could assign students specific paragraphs to preview silently before reading aloud during whole-group instruction. Even this brief preparation time could promote increased confidence and fluency, especially for students who experience anxiety or hesitation around oral reading tasks. One limitation of the present study is the absence of a social validity measure to evaluate participants’ perceptions of reading, particularly their confidence in reading, before, during, and after the intervention. In addition, probes of students’ participation during classroom ELA lessons were not collected, which could have provided valuable insight into how the intervention influenced broader academic engagement. The absence of these probes limits our understanding of whether increased fluency and confidence during the small-group setting generalized to the classroom setting. Future research should explore how incorporating pre-teaching into classroom routines might impact broader classroom behaviors, such as student confidence, self-efficacy, and willingness to participate in academic tasks. Another potential direction for future research is to evaluate whether parents could facilitate pre-teaching at home to support reading development, particularly in cases where review sessions at school are already well-established. Parent training programs could incorporate pre-teaching strategies that emphasize building confidence and readiness for classroom participation.

Although participants volunteered to read in front of their peers to varying degrees across all conditions, all participants consistently volunteered to read the specific passage they practiced during the pre-teaching condition. It is possible that the generally high rates of volunteering to read could be due to the nature of the small group with less proficient readers. Specifically, reading in front of a small group of peers who are also struggling readers may be a discriminative stimulus for the absence of punishment. For example, reading aloud in the small group context reduced the risk of ridicule compared to reading in front of more skilled readers in the typical classroom setting. Practicing a specific passage in the morning that would appear in small group later that day further decreased the risk for social aversive contingencies as well as self-generated aversive contingencies (e.g., negative self-talk about mistakes) by improving reading rate and reducing errors. Another explanation for the generally high rates of volunteering to read can potentially be explained by the presence of the experimenter, who assisted with reading if the participant erred and provided high rates of praise and noncontingent prizes at the end of the small group. This behavior may suggest that students who are typically hesitant to read aloud in front of peers, such as those with low reading confidence or anxiety, could especially benefit from the opportunity to preview reading material beforehand. The increased confidence gained from practicing a passage prior to reading it aloud may reduce avoidance behaviors and support greater classroom participation.

The analysis of WRCM during morning repeated reading probes revealed that all participants demonstrated the highest WRCM on the fourth reading attempt in both conditions. Interestingly, the most substantial increase occurred between the first and second reading attempts. The standard repeated reading procedure recommends three to four re-readings of the same text to produce the most gains in fluency (Therrien, 2004). However, our data suggest that the initial reading, followed by listening passage preview (Begeny et al., 2009), resulted in the largest gain in reading fluency. Following the second attempt, gains in WRCM leveled out and did not produce as large of an increase as the first attempt to the second attempt during both conditions. Future research on this topic could involve examining student preference for how many times they practice a reading passage using the repeated reading approach. Gains in WRCM were minimal beyond the second attempt, and reading the same text repeatedly may have aversive properties to students. Practitioners should consider reducing the number of repeated readings to two attempts, particularly if students are indicating additional repeated readings to be aversive (e.g., excessive pausing, whining, or complaining). However, monitoring reading comprehension with fewer repeated reading attempts is important to ensure gains in reading comprehension from repeated reading are not lost by reducing the number of readings.

Repeated reading passages should be selected on the basis of a child’s assessed instructional reading level (Therrien, 2006). A limitation of this study is that we selected fourth-grade reading passages because that is the reading level students were experiencing in class, not based on reading assessments to determine participants’ current instructional reading level. When using the fourth-grade passages, there were no systematic differences in accuracy or error frequency between pre-teaching and review conditions for all participants. Morgan et al. (2000) found that using reading materials two grade levels above the instructional grade level led to more gains in student reading progress than instructional reading level materials. However, other research indicates that reading accuracy and fluency decreased when elementary students read more advanced text, particularly for struggling readers (Amendum et al., 2017; Morris et al., 2012). Our participants were not meeting benchmark WRCM scores for fourth-grade students during the middle of the school year when reading at their assigned grade level. At the second-grade reading level, all participants showed an overall increase in accuracy and WRCM, which could allow for the detection of differences in improvement across conditions. Unfortunately, a second limitation of this study is that the school year ended, and insufficient data were collected for 2 of 3 participants to conclude that either condition was superior to the other. Future research could address this limitation by conducting individualized reading assessments prior to beginning repeated reading intervention to determine the participants’ current instructional reading level or conferring with the teacher when selecting materials.

In the small group, all participants exhibited the highest WRCM, highest accuracy, and lowest error frequency when reading the specific pre-teaching passage from their morning intervention session. The repeated reading intervention still produced an increase in WRCM for the practiced passage with a delay of approximately 4 hours after the participants experienced the intervention. These results are consistent with Therrien (2004) and indicate that repeated reading is an effective intervention to increase reading fluency for the practiced passage. Interestingly, students performed better during the morning review condition WRCM probes after a delay of approximately 20 hours from the previous small group. Consistent with Ardoin et al. (2018) these findings provide evidence to suggests that having students read a passage just once with error correction, can produce gains in reading fluency in comparison to having no previous interaction with the passage.

In conclusion, this study provides insight into the effects of the arrangement of repeated reading intervention and student preferences for when the intervention is delivered. Participants displayed a strong preference for the pre-teaching condition, volunteered to read consistently when the passage they practice that morning appeared again in the small group session, and read that passage more fluently, which emphasizes the importance of building confidence and readiness for struggling readers when they will be asked to read in front of their peers.

## Supplementary Information

Below is the link to the electronic supplementary material.

Supplementary file1 (DOCX 2902 KB)
