# Supplementary material for: Elementary Students Prefer Extra Reading Practice Before Reading in Front of Peers
Source: Behav Anal Pract. 2025 Sep 22;19(3):1317–28. doi: 10.1007/s40617-025-01099-0 (PMC13638881; doi:10.1007/s40617-025-01099-0)
Supplement: Supplementary file 1 [file 40617_2025_1099_MOESM1_ESM.docx]

**Supplemental Figure 1**

*Accuracy and error frequency during the first WRCM probe in morning intervention sessions.*

*Note. RL = reading level.*

**Supplemental Figure 2**

 *Accuracy and Error Frequency During Small Group Readings.*

*Note. BL = baseline.*

**Supplemental Figure 3**

*Percentage of volunteering to answer comprehension questions.*

*Note. BL = baseline*

**Procedural Fidelity Checklists**

**Afternoon Small Group – Procedural Fidelity Checklist**

Correct materials present (reading passages [one for experimenter, one for participant], timer, rewards)

**___** Correct instructions provided

**___** Experimenter reads the first word if the participant does not begin reading within 3s of saying “Begin”

**___** Experimenter records words read incorrectly by marking the error on their copy

___ Stops timer at one minute and instructs participant to stop reading

___ Records the last word read by marking their copy

**___** Allows participant to select reward

**______ out of 7**

**______________________________________________________________________________**

**Morning Individual Intervention – Procedural Fidelity Checklist**

Correct materials present *(WRCM reading passage [one for experimenter, one for participant], pre-teaching OR review reading passage [one for experimenter, one for participant], timer, colored condition card, rewards)*

**___** Correct condition colored card on display

**___** Correct (pre-teaching or review) instructions provided

**___** Experimenter reads the first word if the participant does not begin reading within 3s of saying “Begin” for initial WRCM probe

**___** Experimenter records words read incorrectly by marking the error on their copy

___ Experimenter stops timer at one minute and instructs participant to stop reading

___ Records the last word read by marking their copy

**___** Experimenter reads (pre-teaching or review) reading passage

___ Participant reads (pre-teaching or review) passage 3x or until they reach mastery criterion

___ Experimenter stops timer at one minute and instructs participant to stop reading

___ Experimenter records the number of times the student read the passage until mastery or reaching the maximum allotted

___ Experimenter helps participant with any words they need assistance with

**___** Allows participant to select reward

**______ out of 13**

**______________________________________________________________________________**
